# Criteria for selecting the Paganin-filter reconstruction parameter in X-ray phase-contrast tomography

**DOI:** 10.1107/S1600577526001694

**Published:** 2026-03-23

**Authors:** Eduardo X. Miqueles, Everton L. de Oliveira, Murilo Carvalho, David M. Paganin

**Affiliations:** a Brazilian Synchrotron Light Laboratory, CNPEM, Campinas, SP, Brazil; b School of Physics and Astronomy Monash University Clayton Australia; Australian Synchrotron, Australia

**Keywords:** tomography, Paganin, noise, algorithm, imaging

## Abstract

The Paganin filter, widely employed in propagation-based phase-contrast X-ray computed tomography with synchrotron light, attenuates phase effects and suppresses high-frequency noise under the assumption that samples have a constant β/δ ratio (these constants define the material’s complex refractive index *n* = 1 − δ + *i*β). While effective in many scenarios, real samples often violate this strong assumption, and the choice of the filter’s free reconstruction parameter is typically made empirically, potentially leading to suboptimal image quality. This work introduces a quantitative, noise-aware criterion for determining a near-optimal filter parameter, thereby removing the need for subjective tuning. We propose two methods for this task. The first method is based on analysis of the noise power spectrum in radial frequency bands, adopting the same partitioning framework as Fourier ring correlation (FRC). By monitoring high-frequency behavior, the proposed approach identifies the trade-off point where resolution enhancement is balanced against noise amplification. This criterion enforces a theoretical lower bound on the FRC computed between two independently split Paganin-filtered projections, where each projection corresponds to a single recorded detector frame, while ensuring effective suppression of high-frequency noise. The second method analyzes the variation of the reconstructed images as a function of the Paganin-filter parameter, identifying the inflection point that represents the balance between noise suppression and image smoothing. Both methods result in an automated parameter selection approach for phase-contrast tomography data analysis that employs the Paganin filter.

## Abstract 2

The development of a criterion for near-optimal selection of the Paganin-filter parameter based on noise power analysis measured via an image’s noise power spectrum is described.

## 1. Introduction

In computational methodologies applied to computed X-ray tomography, especially when performed with a synchrotron light source, it is common for propagation-based phase-contrast measurements to have their phase effects processed through application of the so-called Paganin filter (Teague, 1983 ▸; Paganin *et al.*, 2002 ▸). This filter is based on the assumption that the ratio between the imaginary part (β) and the real decrement (δ) of the complex refractive index *n* = 1 − δ + *i*β is approximately constant across the object. In many experiments, this hypothesis is considered as approximately valid, making the method widely adopted for reconstructing images with adequate quality. However, in experimental practice and under real data acquisition conditions, the sample under investigation will often not be accurately described as having a constant β/δ ratio. In particular, it is common to observe a high heterogeneity of compositions and structural variations, which do not strictly conform to the assumptions of the original ‘single material’ model. Nevertheless, the Paganin filter has become one of the most widely used approaches in synchrotron-based X-ray phase contrast imaging, due in part to its ease of implementation, low computational cost, and ability to produce stable results even under challenging conditions (Gureyev *et al.*, 2014*a* ▸; Nesterets & Gureyev, 2014 ▸; Kitchen *et al.*, 2017 ▸; Gureyev *et al.*, 2017 ▸; Paganin *et al.*, 2020 ▸). Previous studies have addressed the optimization of key parameters in propagation-based phase-contrast imaging, including the regularization parameter in Paganin’s method (Beltran *et al.*, 2018 ▸) and the propagation distance in free-space imaging setups (Gureyev *et al.*, 2004 ▸), highlighting the importance of parameter tuning for accurate phase retrieval.

In addition to attenuating phase artifacts, the filter can reduce part of the noise present in the projections while still maintaining a satisfactory level of fidelity to the original data (Miqueles *et al.*, 2025*a* ▸). This feature makes it particularly attractive for quantitative and qualitative analyses in high-resolution experiments. However, in order for the result to be truly satisfactory, it is essential to appropriately determine the free parameter that is associated with the filter, which controls the degree of smoothing applied. In practice, this choice is often made empirically, being left to the experimental station user, who adopts an *ad hoc* value based on prior experience or preliminary testing.

To eliminate the need for manually choosing a value for the Paganin filter parameter ℓ, there is a clear demand for the development of computational strategies capable of providing the user with a near-optimal value, avoiding reliance solely on subjective judgment or trial-and-error. The goal is to offer a systematic procedure, based on quantitative metrics, that not only automates parameter selection but also reduces the uncertainty associated with this critical processing step. We emphasize that automatic methodologies for finding refractive properties of the material under investigation were already reported in the literature (Eastwood *et al.*, 2011 ▸; Alloo *et al.*, 2022 ▸), although from quite different computational perspectives in comparison with that reported in the present paper. The near-optimal value for the filter parameter, associated with our work, should satisfy different practical requirements simultaneously. On one hand, it should allow a clear separation of the histogram peaks of the obtained projection; this is a criterion that is often used visually, by experienced users, to assess the quality of contrast between regions of the sample. On the other hand, it should be capable of preserving or even enhancing edges and structural features of the object under analysis, thus maximizing the usefulness of the reconstruction for both quantitative and qualitative interpretations.

In this context, the present manuscript addresses the development of a criterion for near-optimal selection of the Paganin-filter parameter ℓ, based on noise power analysis measured via the image’s noise power spectrum (NPS) (Nesterets & Gureyev, 2014 ▸). The proposed approach aligns with methodologies widely used for spatial resolution assessment, such as Fourier ring correlation (FRC) (Saxton & Baumeister, 1982 ▸; Banterle *et al.*, 2013 ▸; Nieuwenhuizen *et al.*, 2013 ▸; Culley *et al.*, 2018 ▸; Tortarolo *et al.*, 2018 ▸) and its three-dimensional variant, Fourier shell correlation (FSC). The central idea is to measure the NPS of the input signal over radial frequency bands, applying a ring partitioning analogous to that used in FRC. This approach is compared with a second method, also proposed in this work, which is based on the standard deviation of the derivative of images with respect to the free parameter ℓ.

This approach offers two main advantages. First, it enables refined monitoring of high-frequency behavior, making it possible to identify the point at which the gain in sharpness is counterbalanced by undesired amplification of noise. Second, it provides a quantitative criterion that naturally aligns with the classical trade-off between noise reduction and fidelity to the original data. Thus, the proposed method not only systematizes parameter selection but also contributes to greater rigor in the analysis and comparison of results obtained in X-ray phase-contrast tomography experiments.

Since the approach developed in this paper may be viewed in the broader context of autofocusing computational imaging systems (Eastwood *et al.*, 2011 ▸), we close this introduction with some general remarks in this regard. Tomography, in general, and X-ray phase-contrast tomography, in particular, is an exemplar of the computational-imaging concept in the sense that the computer forms an intrinsic part of the imaging system, a ‘software lens’ which utilizes a mathematical-physics model of the forward-imaging problem in order to solve the inverse problem of tomographic reconstruction from a set of two-dimensional projection images (Paganin *et al.*, 2004 ▸). At no stage is the value at an individual voxel (*e.g.* a density) directly measured by a pixellated detector, thus the tomographic reconstruction may indeed be viewed as a ‘software lens’. As is the case with the ‘hardware lens’ in the human visual imaging system, the ‘software lens’ associated with Paganin-filtered phase-contrast tomography depends on a single parameter; this single parameter corresponds to a defocus for the former (hardware) imaging system, and a β/δ ratio for the latter (computational/software) imaging system. In analogy to the autofocusing property of the human visual system—whereby we adaptively select an accurate focal length for our eyes’ lenses in order to see an acceptably sharp image—the present paper may be viewed as a computational-imaging procedure whose associated ‘software lens’ (namely Paganin-method phase retrieval followed by tomographic reconstruction) is ‘autofocused’ in the systematic manner referred to earlier in this introduction.

## 2. Methodology

We briefly recall the action of the Paganin filter, which depends on a real parameter ℓ (Paganin *et al.*, 2002 ▸). In the special case of the transport-of-intensity equation (TIE) (Teague, 1983 ▸) that corresponds to a sample composed of a single homogeneous material of possibly variable density, the projection approximation implies that a propagation-based X-ray phase contrast intensity image *f* is mapped into another image *p*_ℓ_ through application of a convolution filter *P*_ℓ_, followed by the logarithm applied to valid pixels, namely 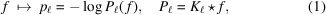
with 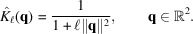
Here the 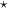 symbol denotes two-dimensional convolution, 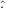 denotes two-dimensional Fourier transformation with respect to Cartesian spatial coordinates (*x*, *y*), **q** ≡ (*q*_*x*_, *q*_*y*_) are the corresponding Fourier coordinates (spatial-frequency coordinates) having units of inverse length, ∥**q**∥^2^ = 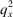 + 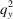, and the filter parameter ℓ has units of squared length. The convolution kernel acts as a low-pass filter in the spatial-frequency domain, attenuating high-frequency components—often associated with noise—while preserving the main structural features of the image. Under the previously stated assumptions, together with the requirements of uniform-intensity coherent plane-wave illumination and a sample-to-detector distance which is sufficiently small for the associated Fresnel number to be much greater than unity, *p*_ℓ_ is approximately proportional to the projected thickness of the homogeneous sample, with this projection being taken along the direction of the optical axis.

It is important to note a distinction with respect to filter-parameter values more commonly used in the literature, where the parameter is often expressed as the dimensionless quotient ℓ_*L*_ = β/δ, with δ and β representing the real and imaginary components, respectively, of the sample’s complex refractive index *n* = 1 − δ + *i*β (Paganin *et al.*, 2002 ▸). In our formulation, we instead adopt the Paganin-filter parameter 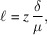
where *z* is the sample-to-detector propagation distance and μ = 4πβ/λ is the linear attenuation coefficient, with λ denoting the X-ray wavelength. This grouping makes explicit the dependence of ℓ on experimental geometry and beam energy. From this definition, it follows directly that 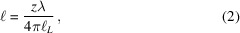
which ensures dimensional consistency of the convolution kernel in equation (1) when expressed in Fourier space. Thus, the uncertainties typically associated with the ratio β/δ are inherently transferred to ℓ through equation (2). In other words, any imprecision in the refractive index-parameters is reflected proportionally in the effective filter parameter ℓ. Once ℓ is determined experimentally or through prior sample- material knowledge, the conversion in equation (2) allows computation of ℓ_*L*_. For completeness, we recall that in practice the input image *f* is the ratio between two other images typically acquired in synchrotron-based X-ray phase contrast tomographic experiments, namely *f* = 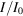, where *I* is the photon count at the detector with the beam passing through the sample and *I*_0_ is the corresponding flat-field image acquired without the sample in the beam path. This normalization step removes the incident beam profile from the data, ensuring that the Paganin-method filtering operates on a corrected intensity distribution representative of the sample alone.

### 2.1. Noise power spectrum criteria

When analyzing the spatial resolution of a single image, it is common practice to split it into two subsets (*e.g.* pixels with even and odd indices) so that the resulting images can be cross-correlated—for instance, via FRC—in order to estimate the resolution limit. In this work, the splitting procedure preserves the statistical nature of the original image, as demonstrated by Rieger *et al.* (2024 ▸), making it valid, for example, for resolution analysis. If we denote the original image by *f*, its split into two complementary images {*u*, *v*} still contains sufficient information regarding the noise structure. This type of image splitting can be implemented in a practical way (Banterle *et al.*, 2013 ▸) with the purpose of determining resolution in microscopy.

The NPS (Nesterets & Gureyev, 2014 ▸) of a noise signal *n* is estimated as the function 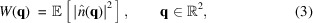
where 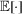 denotes the mathematical expectation of a random variable. When the original image is split into *u* and *v*, the noise content is preserved in each subset, so that the difference *n* = *u* − *v* retains the same statistical noise structure as the original signal.

It is important to emphasize that equation (3) is employed here in the operational sense customary in imaging science. The objects under consideration are finite, discretely sampled detector frames with compact support, rather than idealized infinite stationary random processes. Accordingly, the NPS is estimated via the discrete Fourier transform of a finite noise realization (obtained in this work from the split-image difference), using a periodogram-type estimator. The expectation operator is approximated by averaging over Fourier samples within radial frequency bands, under the usual assumptions of local stationarity and isotropy across the finite field of view. In this discrete finite-dimensional setting, the NPS corresponds to the standard power spectral density (PSD) estimator used in detector and imaging-system characterization [*cf*. Goodman (2000 ▸); Dainty & Shaw (1974 ▸); Nesterets & Gureyev (2014 ▸)], and should not be interpreted as the Fourier transform of an idealized infinite stationary random process.

In order to avoid explicit computation of the expectation operator, we approximate 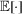 by an average over spatial frequencies, grouping together Fourier coefficients that belong to equivalent frequency domains, under the assumptions of stationarity and isotropy. These equivalent frequency domains are defined as *radial frequency bands*, in exactly the same manner as the rings used in FRC calculations. Here, the radial variable *r* denotes a normalized radial frequency coordinate. More precisely, if ρ = ∥**q**∥ represents the physical radial spatial frequency (with units of inverse length), and 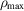 denotes the maximum sampled frequency in the discrete Fourier grid (*i.e.* the Nyquist radius), we define 
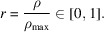
Thus, throughout this work, *r* is dimensionless and represents the relative position within the Nyquist band. In this sense, statements such as 0 < *r* < 1 and choices like *r* ≃ 0.5 refer to fractions of the maximum resolvable spatial frequency (*e.g.* half the Nyquist radius) rather than to absolute spatial frequencies expressed in physical units.

Thus, the NPS can be expressed in terms of frequency bands of Fourier-space radius *r* as 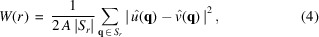
where *S*_*r*_ is the set of Fourier samples (pixels) in the ring corresponding to frequency *r*, |*S*_*r*_| is the number of elements in that set, and *A* is the total physical area of the image in real space. This normalization ensures that *W*(*r*) is expressed in consistent physical units (*e.g.* intensity^2^ × mm^2^) and can be directly compared across different acquisition conditions or detector systems.

*Criterion.* If we take an image *p*, obtained from the Paganin filter with parameter ℓ applied to the original signal *f*, we can state that its split into *u*_ℓ_ and *v*_ℓ_ is such that, as ℓ increases indefinitely, *u*_ℓ_ and *v*_ℓ_ become progressively more similar. This claim follows directly from the definition in equation (1), using the continuity of the logarithmic function, and can be verified with minimal computation. In geometric terms, as ℓ grows, the angle between the images—considered as vectors in a suitable function space (Barrett & Myers, 2004 ▸)—tends toward zero. In physical terms, increasing ℓ increases the strength of the low-pass filtration of the input propagation-based phase contrast image, thereby giving progressively higher relative weight to the (noise insensitive) lowest-order Fourier frequencies in the power spectrum of the measured signal. Naturally, this implies that the correlation of *u*_ℓ_ and *v*_ℓ_ approaches 1 in a monotonic fashion.

Mathematically, this behavior is made explicit through the FRC between *u*_ℓ_ and *v*_ℓ_, expressed via the curve *c*_ℓ_ as a function of the radial frequency band *r* in Fourier space, namely, 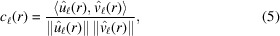
where ℓ is fixed for the computation of *c*_ℓ_(*r*) but may vary when comparing results for different filter strengths. Above, ∥ · ∥ denotes a norm induced by an inner product 〈·,·〉 in the function space *L*_2_ (Barrett & Myers, 2004 ▸).

**Figure 1:**
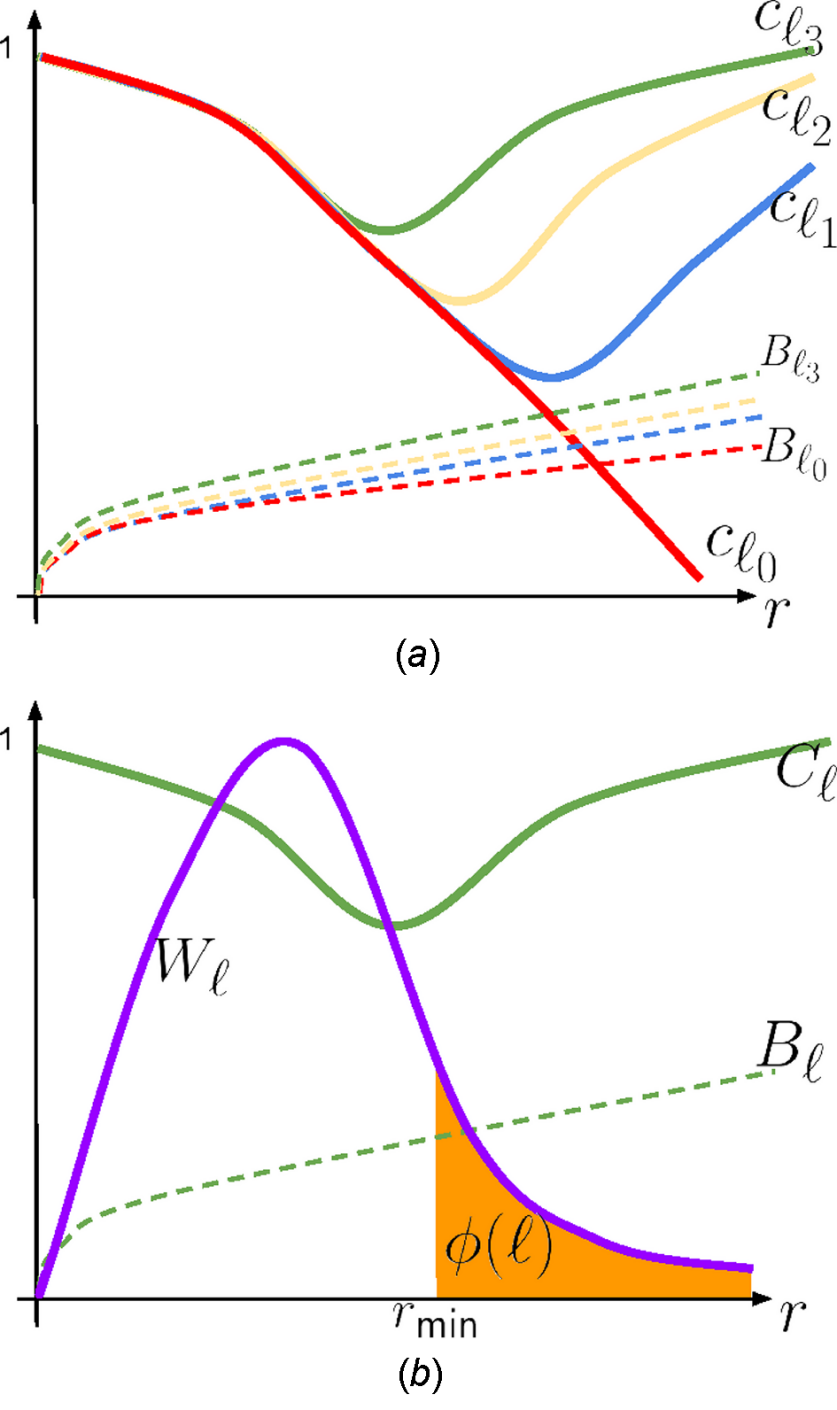
(*a*) Typical shape of correlation curves *c*_ℓ_(*r*) for different values of the Paganin-filter parameter ℓ, with ℓ_0_ < ℓ_1_ < ℓ_2_ < ℓ_3_. Each curve 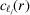 must always remain above its corresponding lower bound 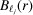. (*b*) NPS curve, given by *W*_ℓ_, as a function of ℓ. As ℓ increases, the correlation curve *C*_ℓ_ always remains above *B*_ℓ_, monotonically tending toward 1, while the tail of *W*_ℓ_ monotonically decays toward zero.

It was demonstrated recently (Miqueles *et al.*, 2025*b* ▸) that, for any fixed ℓ, the curve *c*_ℓ_(*r*) satisfies a theoretical lower bound *B*_ℓ_(*r*) (see Miqueles *et al.*, 2025*b* ▸), which depends parametrically on ℓ,
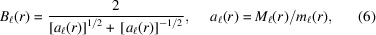
where *M*_ℓ_(*r*) and *m*_ℓ_(*r*) denote, respectively, the maximum and minimum magnitudes of the Fourier coefficients of the split images over the ring *S*_*r*_. In other words, for all admissible radial frequencies *r*, one has *c*_ℓ_(*r*) ≥ *B*_ℓ_(*r*). As ℓ increases, the strength of the low-pass filtering increases, leading to progressively higher correlation between the split images across all frequency bands. Consequently, for each fixed *r*, the value *c*_ℓ_(*r*) increases and tends toward 1, corresponding to perfect correlation (*i.e.* the horizontal line *c* = 1 in the limit of very strong filtering). This qualitative trend is illustrated schematically in Fig. 1 ▸(*a*).

From this perspective, *u*_ℓ_ and *v*_ℓ_ behave as function-space vectors whose mutual angle decreases toward zero in the limit ℓ → ∞, making them nearly linearly dependent. The trade-off we seek is to determine the point at which we should stop increasing ℓ, so that this angle does not vanish entirely. This is where the NPS-based criterion comes into play, providing a quantitative way to balance how much this angle is allowed to close. Proposition 1[Statement proposition1] summarizes the algorithmic methodology we aim to adopt.

Proposition 1 Let *c*_ℓ_(*r*) denote the split-image FRC curve defined in equation (5), and let *B*_ℓ_(*r*) be the corresponding consistency lower bound given in equation (6). Assume that, for each fixed ℓ, the consistency inequality 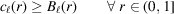
holds (*cf*. Miqueles *et al.*, 2025*b* ▸). Define 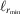 as the smallest filter parameter ℓ such that the normalized high-frequency noise level satisfies 
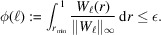
Then 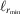 provides a data-driven, noise-aware choice of Paganin-filter strength that enforces the prescribed suppression of high-frequency noise while remaining consistent with the split-image FRC bound.

The functional ϕ(ℓ) in Proposition 1[Statement proposition1] measures the residual normalized noise power in the high-frequency band 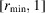. Throughout this work we set 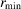 = 0.5 as a conservative default, and we select 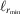 as the smallest ℓ satisfying ϕ(ℓ) ≤ ε. For convenience, we restate this condition as 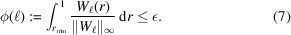
The NPS curve given by *W*_ℓ_, as a function of the Paganin-filter parameter ℓ, is shown in schematic form in Fig. 1 ▸(*b*). As the value of ℓ increases, the correlation curve *C*_ℓ_ always remains above *B*_ℓ_ [ensuring consistency, as expected (Miqueles *et al.*, 2025*b* ▸)], monotonically tending toward unity while the tail of *W*_ℓ_ monotonically decays toward zero, so that the integral ϕ(ℓ) given by equation (7) becomes progressively smaller.

We observe that the 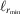 such that ϕ(ℓ) ≤ ε is obtained by a straightforward bisection method which, thanks to the monotonic behavior of ϕ(ℓ) with respect to ℓ, typically converges in only a few iterations. As defined, the near-optimal value of ℓ derived from the NPS criterion depends explicitly on 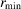. In practical terms, the larger 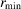 is, the lower the resulting ℓ must be; conversely, for smaller 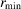 the solution tends toward higher values of ℓ. Hence, there is a conceptual trade-off between committing to a single value or to an *interval* for ℓ, reflecting our uncertainty about where the ‘high-frequency’ range should begin. We therefore opt to specify a *fuzzy* interval of near-optimal values, for example ℓ_0.6_ < ℓ < ℓ_0.5_, or, more generally, 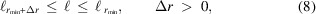
where the span Δ*r* can be chosen in accordance with the spectral resolution (*i.e.* the ring partition) employed in computing *W*_ℓ_(*r*) and *c*_ℓ_(*r*). In principle, there may exist an additional criterion—defined via a suitable cost function—that selects a single ℓ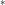 within this interval.

### 2.2. Standard deviation and correlation criteria

**Figure 2:**
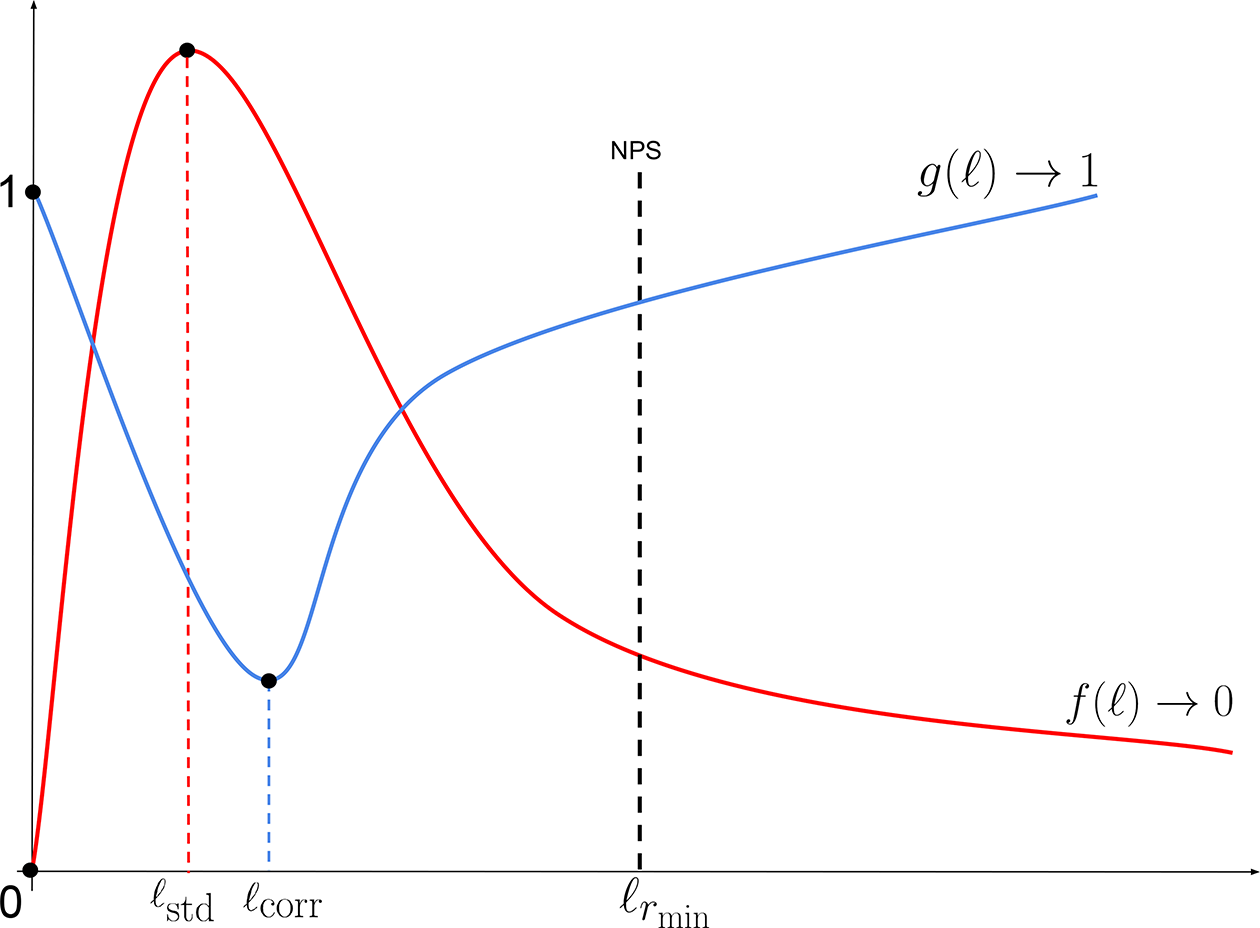
Typical shape of the curves {*f*, *g*} in equation (9), exhibiting the optimal point within the interval 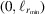.

To obtain a second criterion for evaluating the near-optimal parameter ℓ, we now consider two Paganin-filtered images *p*_ℓ_ and *p*_ℓ+*h*_, where *h* denotes a suitable step size in the filter parameter. It is natural to expect that, for sufficiently small variations of *h*, the difference between the images *p*_ℓ_ and *p*_ℓ+*h*_ remains small for certain values of ℓ. In this sense, as ℓ increases, noise is progressively more strongly suppressed and the difference *p*_ℓ_ − *p*_ℓ+*h*_ mainly contains only numerical noise associated with the subtraction of the two images. On the other hand, for small values of ℓ, the same difference preserves structural details of the object. Thus, for a fixed step *h*, we define the criterion function 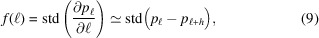
which measures the degree of noise suppression described above. In other words, the larger the standard deviation (std) between two images, the lower their mutual correlation. Based on this reasoning, we search for an optimal point of *f* in order to define a near-optimal parameter ℓ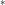. In Appendix *A*[App appa] we demonstrate that *f*(ℓ) is monotonically decreasing for large values of ℓ, but necessarily possesses a maximum in the interval (0, ∞). To restrict the search space of this maximum, we focus on the interval 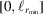, where 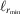 is the value obtained by the NPS-based criterion described in the previous section. Consequently, we may formulate the following optimization problem, 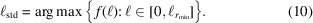
A schematic illustration of this function is presented in Fig. 2 ▸, where the elbow of *f* is clearly visible, corresponding to the near-optimal point ℓ_std_.

Alternatively, as a counterpart to the standard deviation function, we may define another merit function via 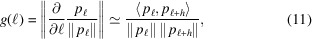
where (as previously mentioned) ∥ · ∥ denotes a norm induced by an inner product 〈·,·〉 in the function space *L*_2_. In Appendix *B*[App appb] we provide a detailed derivation of the approximation in equation (11). Since *g* is a measure of the correlation between consecutive images, it is expected, in the same spirit as the function *f*, that *g* possesses a local minimum. Indeed, as *g* → 1 when ℓ → 0 and *g* → 1 as ℓ → ∞, it is natural to expect that *g* admits a local minimum in the interval (0, ∞). A schematic plot of the function *g* is illustrated in Fig. 2 ▸, where the near-optimal value ℓ_corr_ is obtained through 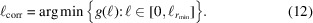


## 3. Experiments

**Figure 3:**
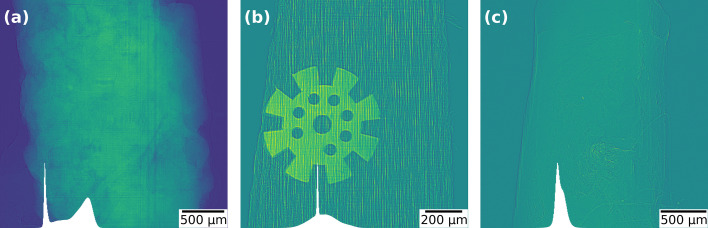
Representative projection frames illustrating the versatility of the proposed approach: (*a*) a natural rock sample, (*b*) a wooden toothpick with a micro gear inside, and (*c*) a thin biological tissue (soft murine cardiac tissue). Data were collected at the MOGNO beamline of the Sirius synchrotron light source.

The numerical experiments consider the images shown in Fig. 3 ▸. The first image (*a*) represents a rock sample, whereas the second image (*b*) corresponds to a wood specimen in which a titanium object is embedded. The third image is a biological soft tissue (soft cardiac tissue from a murine sample). All datasets were acquired at an X-ray energy of 22 keV, with effective pixel size τ = 1.33 µm and sample-to-detector propagation distance of *z* = 0.08 m for sample (*a*); τ = 0.6 µm and *z* = 0.4 m for sample (*b*); and τ = 1.22 µm, *z* = 0.15 m for sample (*c*). In Fig. 3 ▸, the histogram of each image is displayed in the lower-left corner of its respective panel, allowing one to visualize the separation (or overlap) between the intensity distributions. The images shown correspond to the normalization of the raw data, *i.e.*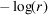 with *r* = *I*/*I*_0_. According to equation (1), these images are equivalent to applying the Paganin filter with ℓ = 0. From a practical standpoint, the goal is to enhance contrast via the regularization operator in equation (1) while simultaneously minimizing the intrinsic measurement noise. This contrast gain typically facilitates subsequent data-analysis tasks such as segmentation and phase quantification, since image quality can be assessed, for example, by the separation of histogram peaks and by improved edge definition. Accordingly, ℓ is expected to be chosen so as to promote such separation without introducing an excessive loss of high-frequency detail. In what follows, we report and compare the near-optimal values obtained by the proposed criteria (based on NPS, standard deviation, and correlation), and discuss the visual and quantitative impact of this choice for the studied samples.

**Figure 4:**
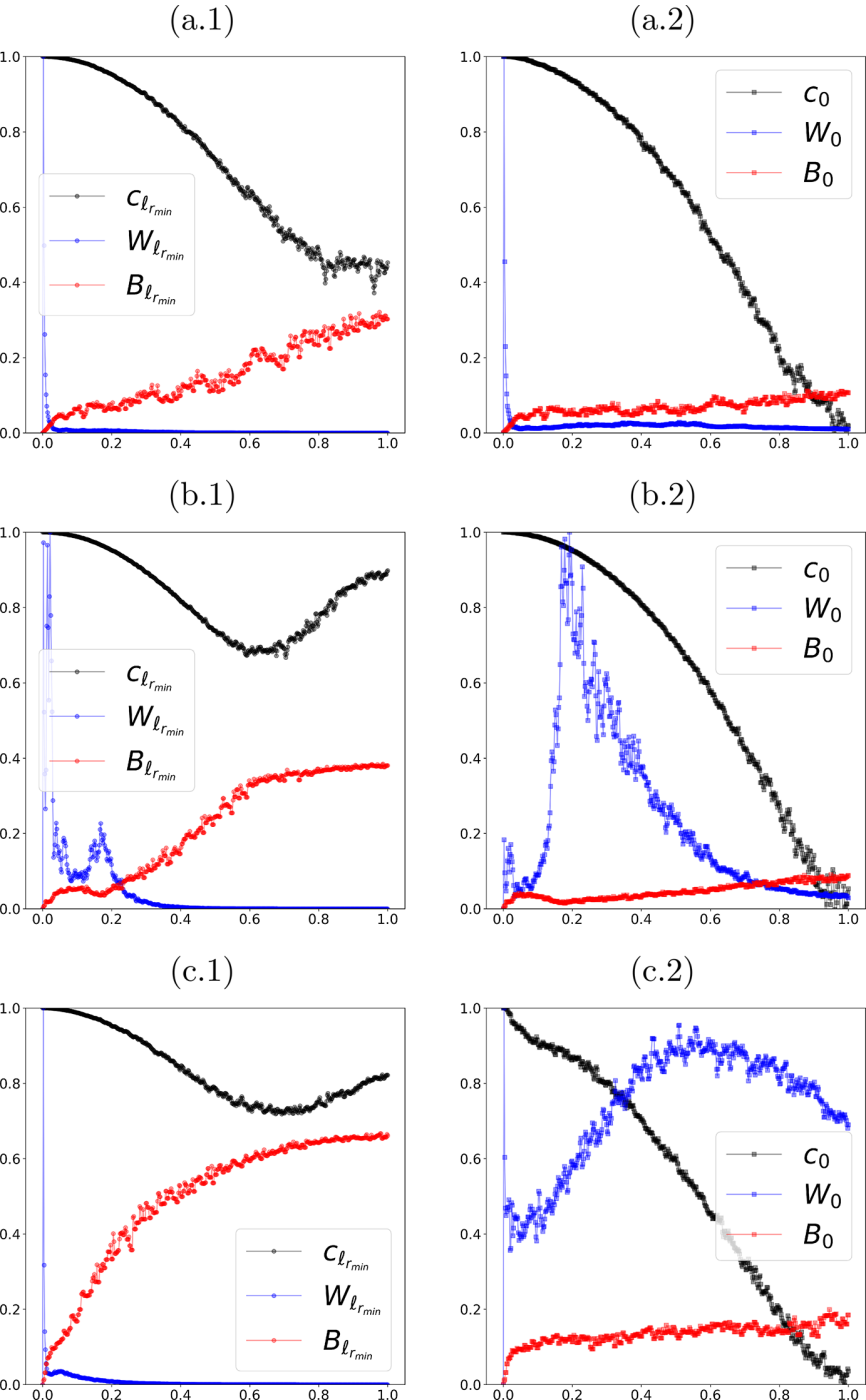
Curves {*c*_ℓ_, *W*_ℓ_, *B*_ℓ_} corresponding to the FRC, the NPS, and the consistency lower bound, respectively, for each sample (*a*), (*b*) and (*c*). As ℓ increases, *c*_ℓ_ exceeds *B*_ℓ_ (consistency), while *W*_ℓ_ approaches zero at high spatial frequencies.

We begin by presenting the behavior indicated in Figs. 1 ▸(*a*) and 1 ▸(*b*), now using the real data. Fig. 4 ▸ shows the empirical behavior of the FRC curves *c*(*r*) given by equation (5), computed via the same ring-wise radial partition in frequency space adopted in the theoretical analysis. In this figure we report, for reference, the value 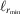 obtained with 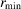 = 0.5 (see Table 1 ▸), as well as the unfiltered case ℓ = 0. This allows a side-by-side comparison of the three quantities of interest {*c*_ℓ_, *W*_ℓ_, *B*_ℓ_}: the FRC curve, the NPS curve, and the consistency lower bound. As predicted by theory (see the previous section), as ℓ increases, the curve *c*_ℓ_(*r*) rises and departs from *B*_ℓ_(*r*) across the entire frequency range, satisfying the consistency condition *c*_ℓ_(*r*) > *B*_ℓ_(*r*) (Miqueles *et al.*, 2025*b* ▸). In parallel, the NPS *W*_ℓ_(*r*) exhibits progressive suppression at high spatial frequencies, with its ‘tail’ becoming flatter and of smaller magnitude. For a fair comparison, the curves are obtained by averaging over each ring *S*_*r*_ and are displayed on the same discretization of *r* ∈ [0, 1]; when appropriate, *W*_ℓ_ is normalized by a suitable norm so that different scales are directly comparable across samples.

By requiring *W*_ℓ_(*r*) to decay smoothly toward values close to zero from 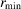 = 0.5 onward—that is, by enforcing the criterion ϕ(ℓ) ≤ ε in equation (7)—we ensure that high-frequency noise contributions are effectively suppressed without unduly penalizing the lower-frequency components that contain the relevant structural information. In this way, the selected 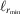 simultaneously satisfies (i) *c*_ℓ_ > *B*_ℓ_ (ensuring internal consistency) and (ii) high-frequency noise mitigation, thereby characterizing the near-optimality of the parameter under the proposed criterion. We further note that the choice of 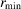 can be tuned according to the spectral resolution of the ring partitioning or the expected scale of meaningful contrast variations, defining a ‘fuzzy’ interval of plausible ℓ values that adapts naturally to the experimental conditions and detector sampling [*cf*. equation (8)]. This flexibility makes the procedure robust to moderate variations in signal-to-noise ratio and acquisition geometry.

We observed that the bisection method used to determine the near-optimal value 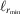 typically takes about 100 s for a search over the interval [0, 10^8^], which we consider acceptable given the large dynamic range of the parameter. By recursively halving the search range into smaller subintervals, the procedure consistently converges to an appropriate ℓ value within a few dozen iterations, without the need for manual tuning. In practice, the algorithm is highly stable and reproducible across datasets of different sizes and exposure levels, reinforcing the reliability of the near-optimal selection rule.

**Figure 5:**
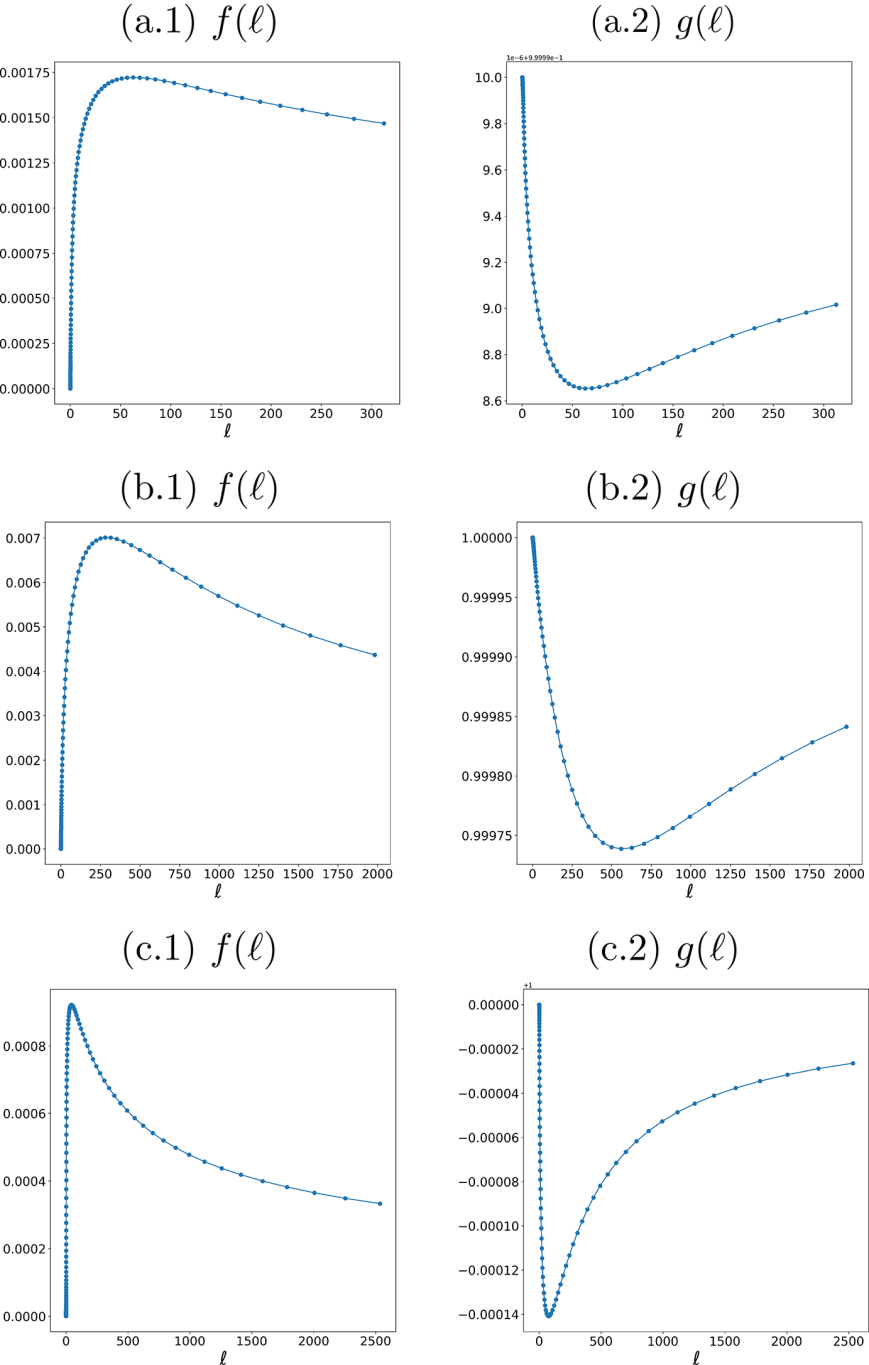
Curves {*f*, *g*} defined in equations (9) and (11) [for samples (*a*), (*b*) and (*c*)], evaluated over log-spaced grids of 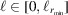. Column (1) shows *f*(ℓ) and column (2) shows *g*(ℓ). The maximum of *f* and the minimum of *g* yield the near-optimal values ℓ_std_ and ℓ_corr_, respectively.

To obtain the values of ℓ_std_ and ℓ_corr_, we construct the curves *f* and *g* defined in equations (9) and (11), respectively, over a logarithmically spaced set of ℓ values (see Fig. 5 ▸). In practice, we compute *f*, *g* on a mesh of 128 points split into eight logarithmic subintervals, which provides both sufficient density and computational efficiency to reveal the maxima and minima of interest. This layout resolves the intricate peak–valley structure of *f* and *g* that a naive linear sampling would often miss or undersample—especially near very small or very large ℓ, where the system response changes rapidly (by several orders of magnitude). Building the curves *f*, *g* amounts to a single computational loop that depends only on the normalized frame *r* = *I*/*I*_0_: for each ℓ, we apply the filtering operations to obtain *p*_ℓ_ and *p*_ℓ+*h*_, as specified in equations (9) and (11). For our datasets, this loop takes approximately 2 min on a standard CPU workstation, which we regard as a reasonable computational cost for selecting a near-optimal ℓ. Moreover, the computation is embarrassingly parallel (Breen *et al.*, 2008 ▸) and can be further accelerated on multi-core or GPU hardware if required for batch processing or high-throughput analysis.

**Figure 6:**
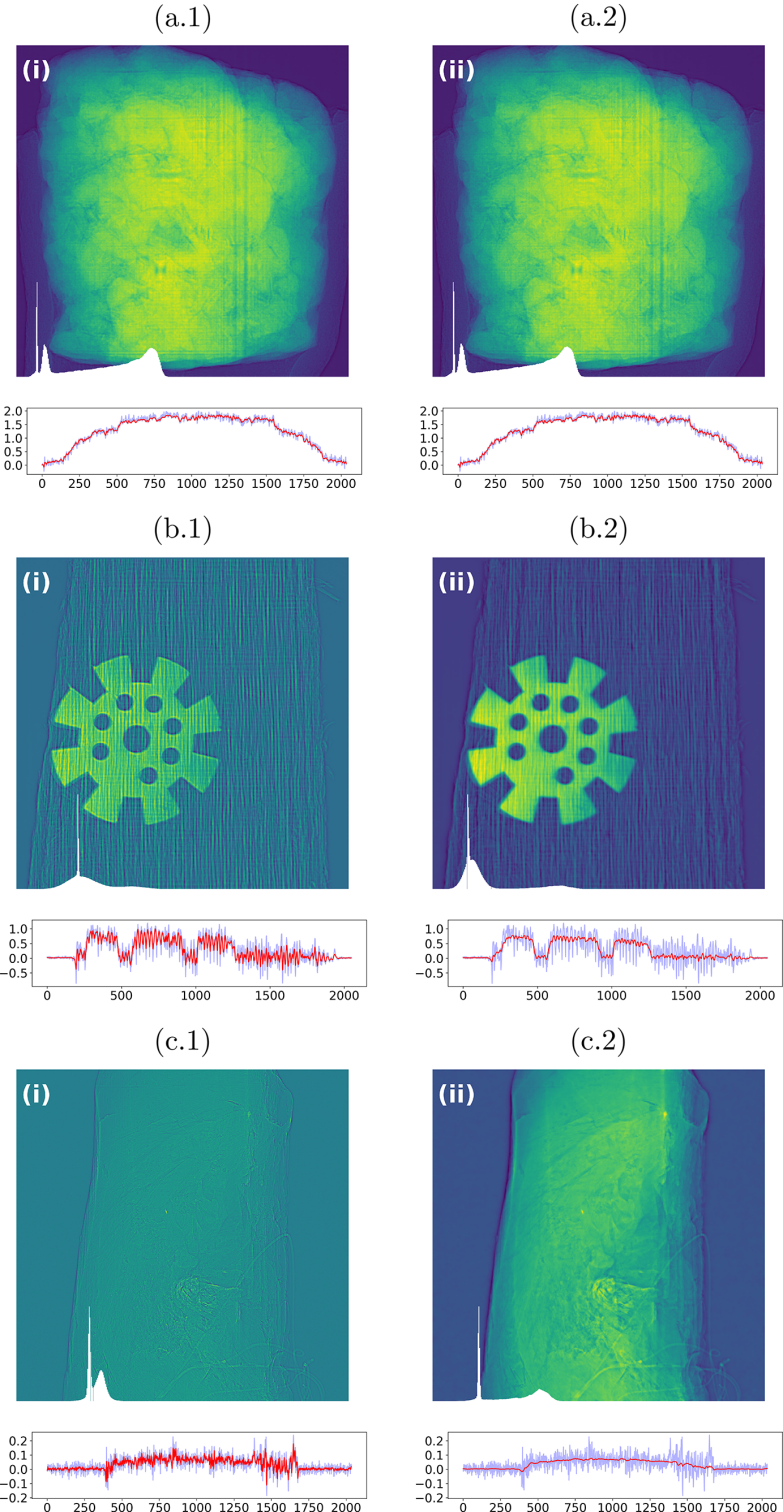
Paganin-filtered images corresponding to the parameter values ℓ_std_ and 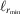 listed in Table 1 ▸. For each image, the central line profile is shown beneath the panel, and the corresponding histogram appears in the lower-left corner, aiding visual assessment of contrast and noise. Accompanying line-profile plots show the noisy unfiltered profile (blue line) and the filtered profile obtained using the near-optimal proposed filter value (red line).

For sample (*a*), the Paganin-filtered images obtained with the parameters ℓ_std_ and 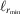 [*cf*. equation (1)] are shown in Figs. 6 ▸(*a*.1) and 6(*a*.2), respectively. The central sectional line profiles are plotted beneath each panel, extracted along the midline of the image to enable a direct, pointwise comparison of edge sharpness and background fluctuations. For sample (*b*), the corresponding results are displayed in Figs. 6 ▸(*b*.1) and 6(*b*.2). Sample (*c*) is shown in Figs. 6 ▸(*c*.1) and 6(*c*.2). In every panel, the histogram of the displayed image appears in the lower-left corner and can be directly compared with the raw-data histograms in Fig. 3 ▸. Consistent with the proposed selection criteria, the histograms exhibit increased separability between intensity modes after filtering, which facilitates downstream segmentation and thresholding after tomographic reconstruction—a key practical advantage of the Paganin-filter approach, particularly in automated workflows.

In our data, 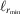 yields stronger high-frequency suppression, while ℓ_std_ preserves slightly more fine detail, leading to a balanced trade-off between denoising and edge retention. The overlaid line profiles further demonstrate a clear reduction in background variance and smoother plateau regions, with only modest attenuation of steep transitions. Overall, these results corroborate the proposition that the chosen parameter values significantly improve image contrast and uniformity while mitigating high-frequency noise, thereby enhancing interpretability and robustness for subsequent quantitative analysis and feature extraction.

## 4. Discussion

It is worth recalling that the physical meaning of the Paganin-filter parameter ℓ is to remove phase-contrast artifacts so as to recover a projected thickness of the specimen (Paganin *et al.*, 2002 ▸). In the single-distance, homogeneous TIE formulation, this corresponds to choosing ℓ to be large enough such that the phase term is effectively retrieved and the resulting image behaves as a projected thickness (up to a proportionality factor). In practice, however, such values of ℓ are typically very large and the associated low-pass kernel 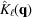 = 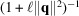 becomes extremely aggressive at high spatial frequencies, producing very smooth images and, consequently, a noticeable loss of spatial resolution relative to the measured data. For instance, for sample (*a*) the thickness-recovery choice yields ℓ = 2793 m^2^, whereas for sample (*b*) it yields ℓ = 56810 m^2^. These numbers can also be determined numerically via a simple bisection search over ℓ for which the filtered, log-normalized image *p*_ℓ_ attains a positive (or nonnegative) signal profile along a representative line section, *i.e.* one increases ℓ while monitoring the filtered profile until a prescribed positivity criterion is met. While this procedure is physically meaningful for projected-thickness recovery, the resulting ℓ values are orders of magnitude larger than those in Table 1 ▸; by design they prioritize strong phase-contrast-fringe removal over resolution preservation and are therefore excluded from the comparative analysis in this study.

**Figure 7:**
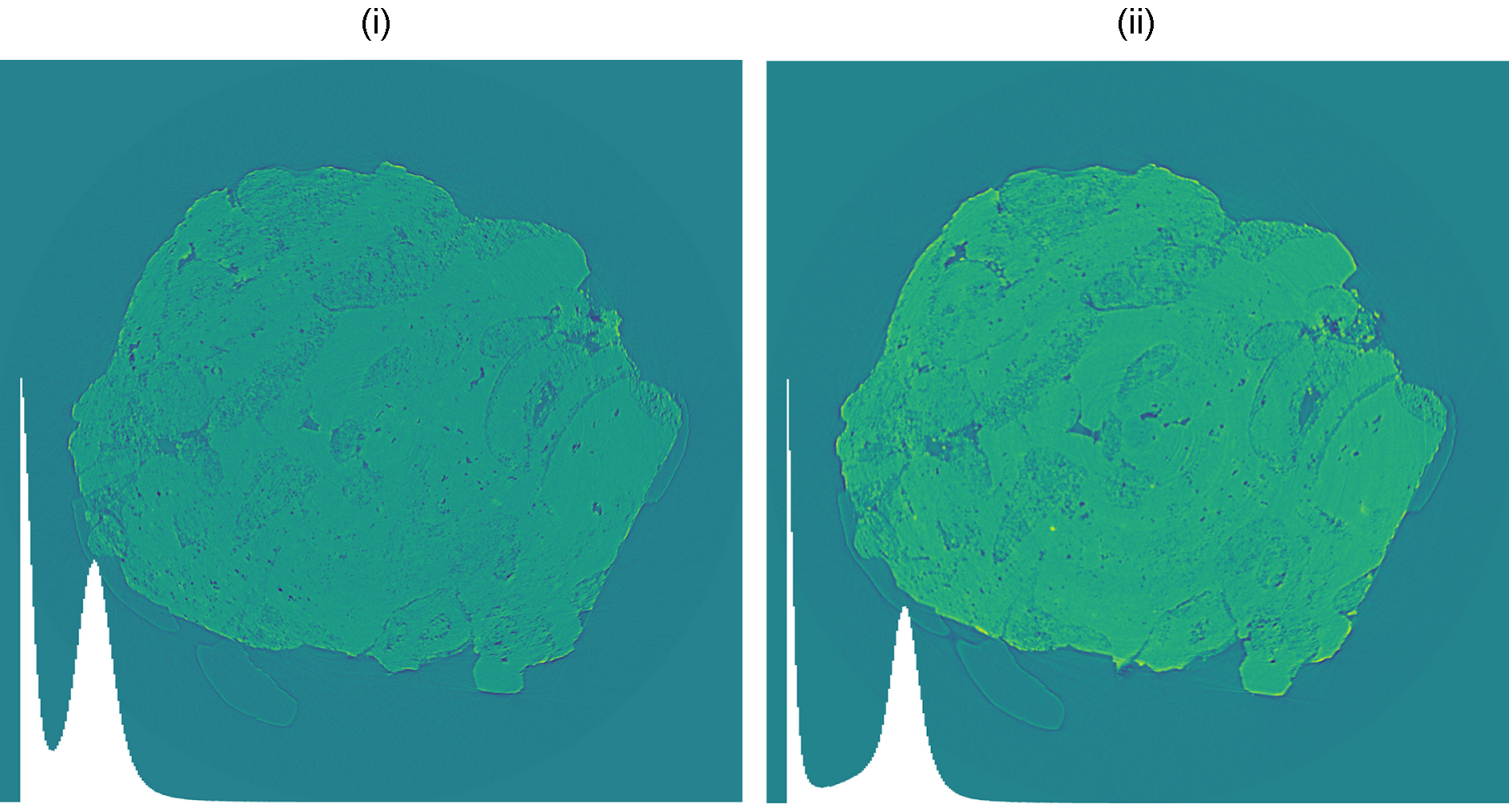
Tomographic reconstructions for sample (*a*), without (i) and with (ii) the Paganin filter using the near-optimal value ℓ_std_, which improves histogram separation.

A second point concerns how the parameter values reported in Table 1 ▸ impact the tomographic reconstruction itself. It is well known that reconstruction algorithms—whether analytical (*e.g.* filtered backprojection) or iterative—introduce numerical artifacts such as streaks, ring artifacts, residual beam-hardening signatures, and edge over-/undershoots. These effects can distort the final histogram of the reconstructed slices or volumes, thereby hampering subsequent segmentation and quantitative analysis. Encouragingly, with the numerical selection strategy proposed here, the reconstructions preserve essentially a similar level of histogram separability achieved at the projection stage: the high-frequency noise is mitigated without excessive blurring, and the class boundaries in intensity space remain well separated after backprojection. This behavior is illustrated in Fig. 7 ▸, where we compare two reconstructions obtained from identical data: (i) without the Paganin filter and (ii) with the filter using a near-optimal ℓ. In addition to the visual impression, the improvement is corroborated by the tighter histograms and smoother midline profiles, which are consistent with our NPS- and correlation-based criteria. In short, the selected ℓ balances phase-artifact suppression and resolution retention so that the advantages observed at the projection level carry through to the reconstructed images, facilitating robust thresholding and phase segmentation downstream.

Another way to quantify the benefit of using the near-optimal values of ℓ is through computation of the standard deviation relative to the mean intensity for each of the filtered images. In practice, for a selected region of interest (ROI) within the reconstructed image, we evaluate a local deviation map that represents, over a coarse grid, the spatial distribution of intensity fluctuations. This map provides an intuitive visual indicator of the performance gain obtained when using each of the parameter values 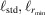. The local deviations quantify how much the gray levels vary around their local mean, thus serving as a measure of local contrast (or texture) rather than image sharpness. In homogeneous areas—where intensity variations are small—the local deviation map exhibits low values, indicating smooth regions with little texture. Conversely, in regions containing edges, fine structural details, or strong noise components, the deviation values increase, yielding a higher contrast response.

**Figure 8:**
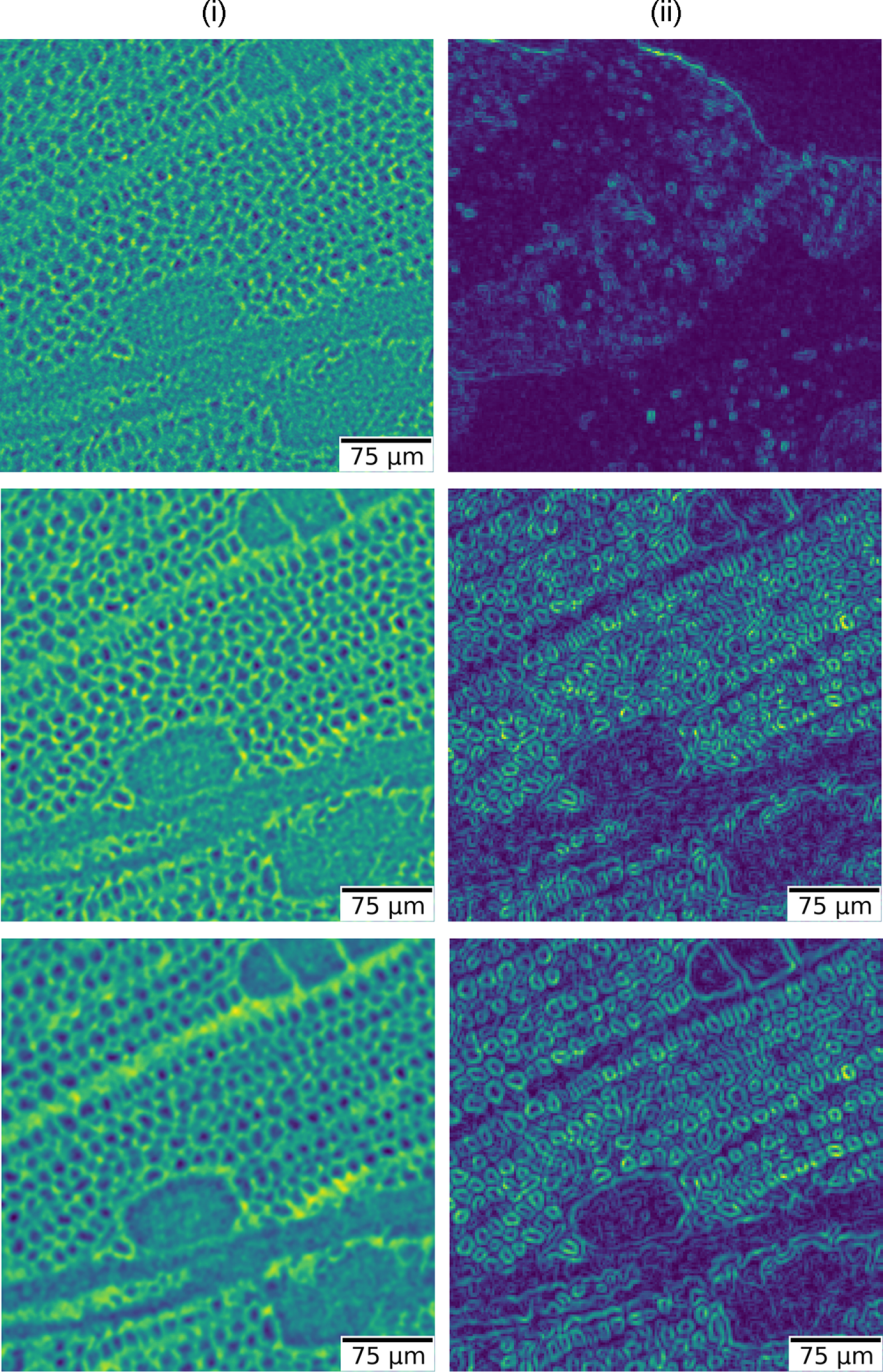
Local-contrast assessment for near-optimal Paganin-filter parameters using sample (*b*) as a reference. Column (i) shows ROIs reconstructed with ℓ = 0, ℓ_std_, and 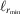, respectively; column (ii) gives corresponding blockwise local-contrast maps (standard deviation relative to the local mean), where brighter tones indicate larger intensity dispersion. Consistently, ℓ = 0 gives lower contrast, 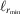 yields higher contrast with stronger blur, and ℓ_std_ provides the best compromise for downstream quantitative analysis.

These local contrast maps are displayed in Fig. 8 ▸. In this figure, column (i) corresponds to the reconstructed regions of interest obtained using ℓ = 0, ℓ_std_, and 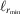 for sample (*b*). A gradual increase in image blur can be visually perceived from above to below. In column (ii), we present the corresponding local contrast maps derived from column (i), respectively. The color scale of these maps highlights the degree of local intensity dispersion, where brighter tones indicate larger fluctuations. As observed, the case ℓ = 0 produces an image with lower contrast and weaker local variations, whereas 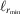 results in stronger local contrast at the expense of increased blurring due to the higher suppression of high frequencies. Between these two extremes, ℓ_std_ offers the most balanced compromise, effectively enhancing local contrast while maintaining acceptable sharpness—thereby facilitating quantitative image analysis, segmentation, and texture-based classification in subsequent processing stages.

The overall trend of Fig. 8 ▸ remains consistent: the near-optimal parameter 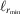 increases the visual separability of features and enhances the apparent texture contrast, while ℓ_std_ provides an intermediate response that is particularly suitable for quantitative tomographic reconstruction workflows, where both noise control and structural fidelity are required. Together, these observations reinforce the robustness and general applicability of the proposed selection criterion across samples of different physical nature and contrast regime.

A complementary view is provided by the local contrast analysis summarized in Fig. 8 ▸. Because the maps are based on the standard deviation relative to the local mean (a blockwise coefficient of variation), they respond primarily to textural fluctuations rather than to high-frequency edge sharpness. This distinction is useful in practice: it decouples *perceived* contrast improvement from resolution loss, making explicit the trade-off negotiated by the Paganin-filter parameter. In our datasets, 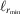 reliably boosts local contrast in heterogeneous regions (*e.g.* porous or fibrous textures), while ℓ_std_ prevents over-smoothing of steep transitions, preserving diagnostically relevant edges. As a working guideline, we therefore recommend choosing the parameter ℓ_std_ when edge delineation is critical (*e.g.* metrology or thin-feature quantification) and 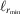 when robust texture separation or histogram mode separability is the priority (*e.g.* automated segmentation pipelines).

From an implementation standpoint, both selection procedures are reproducible and computationally lightweight. The NPS-based bisection is monotone in ϕ(ℓ) and needs only ring-wise frequency statistics; the {*f*, *g*} scans are embarrassingly parallel over a log-spaced grid and depend solely on applying *p*_ℓ_ and *p*_ℓ+*h*_. In practice, this design makes the selection step easy to cache and to amortize across batches of projections or slices. We note three limitations that suggest avenues for future refinement: (i) non-stationary noise and detector anisotropy may violate the ‘isotropic ring’ assumption underlying *W*_ℓ_(*r*); (ii) strong ring or beam-hardening artifacts can bias both the NPS tail and the local-contrast estimates unless pre-corrected; and (iii) the effective modulation transfer function (MTF) of the acquisition system is not explicitly modeled here. Incorporating an MTF-aware normalization or adaptive (angle-dependent) ring partitioning could further sharpen the link between ϕ(ℓ) and visually perceived resolution. Despite these caveats, the observed agreement between the NPS-consistency criterion and the {*f*, *g*} extrema across diverse specimens (rock, plant, and soft tissue) supports the robustness and generality of the approach.

The near-optimal Paganin-filter parameter introduced in this work should not be interpreted as relating to an intrinsic physical property of the sample. Rather, it provides an effective, data-driven estimate of the regularization strength required by the Paganin filter when prior knowledge of the material parameters is incomplete or uncertain. In the homogeneous-material approximation underlying the Paganin method, the filter parameter is directly related to the ratio β/δ, which itself encodes assumptions regarding composition, density, and beam energy. In practice, however, these quantities are often only approximately known, and small deviations can significantly affect the balance between noise suppression and spatial resolution. Important context for the preceding statement is provided by the well known tradeoff between noise and spatial resolution, both in general imaging-physics terms (den Dekker & van den Bos, 1997 ▸; Neifeld, 1998 ▸; van den Bos & den Dekker, 2001 ▸) and in the specific setting of inverse problems in X-ray propagation-based phase-contrast imaging (Gureyev *et al.*, 2014*b* ▸; de Hoog *et al.*, 2014 ▸; Gureyev *et al.*, 2015 ▸; Gureyev *et al.*, 2016 ▸; Gureyev *et al.*, 2020 ▸). The proposed criterion can be viewed as compensating for the previously mentioned uncertainties by selecting a reconstruction parameter that stabilizes the phase-retrieval process while remaining consistent with the physical constraints of the model. Although the resulting parameter may be interpreted in physical terms of an effective strength associated with the degree of propagation-based X-ray phase contrast (Snigirev *et al.*, 1995 ▸; Cloetens *et al.*, 1996 ▸; Wilkins *et al.*, 1996 ▸), its primary role is computational, *i.e.* to adapt the regularization to the data in a physically meaningful way, without introducing additional free parameters or significant computational overhead.

The sensitivity of the proposed parameter-selection criterion to experimental conditions such as noise level, exposure time, and detector binning follows naturally from its formulation. Changes in exposure time or photon flux directly affect the signal-to-noise ratio of the acquired projections, which is captured by the FRC computed between independently split images. In photon-starved regimes, increased noise leads to a faster decay of the FRC at high spatial frequencies, resulting in the selection of a more conservative regularization parameter that prioritizes noise suppression. Conversely, in high-flux or long-exposure conditions, improved photon statistics allow higher spatial frequencies to be preserved, yielding a weaker regularization. Detector binning produces a similar effect by modifying the effective noise level and frequency content of the data. As a result, while the proposed criterion does not explicitly model these acquisition parameters, it adapts implicitly to different experimental regimes through the data-driven FRC constraint, remaining applicable across a wide range of synchrotron imaging conditions.

Although the proposed criterion is formulated within the homogeneous-material assumption underlying the original form of the Paganin method, its data-driven nature allows it to remain meaningful in more general imaging scenarios. In objects with spatially varying β/δ ratios or heterogeneous material composition, the selected parameter can be interpreted as an effective regularization that balances noise suppression and resolution across the field of view rather than as a locally exact physical quantity. In this sense, the method does not resolve material heterogeneity, but provides a stable global parameter choice that is robust to moderate deviations from homogeneity. In multi-distance phase-retrieval setups, the criterion may be applied independently at each propagation distance, or used to inform a consistent parameter selection when combining information from multiple distances. While dedicated multi-material or multi-distance methods remain necessary for quantitative material discrimination, the proposed approach offers a complementary and computationally inexpensive tool for parameter selection in practical experimental conditions.

## 5. Conclusion

We have introduced two quantitative and complementary criteria to select a near-optimal Paganin-filter parameter for propagation-based phase-contrast X-ray tomography, replacing empirical tuning with a reproducible and noise-aware procedure. The first criterion relies on the noise power spectrum evaluated over FRC-style radial bands and enforces both internal consistency—via the lower bound *B*_ℓ_(*r*) with *c*_ℓ_(*r*) > *B*_ℓ_(*r*)—and high-frequency noise suppression through the integral constraint ϕ(ℓ) 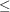

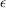
. The second criterion exploits the extremal behavior of two merit functions derived from finite-difference approximations to ∂*p*_ℓ_/∂ℓ: the standard-deviation curve *f*(ℓ) (maximized at ℓ_std_) and the correlation curve *g*(ℓ) (minimized at ℓ_corr_). Across rock, plant, and soft-tissue datasets, the three estimates (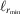, ℓ_std_, ℓ_corr_) produced reconstructions with improved histogram separability, lower background variance, and preserved structural interpretability.

From a practical standpoint, 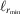 reliably achieves stronger high-frequency suppression, which benefits segmentation and texture separation, while ℓ_std_ typically preserves slightly sharper edges and fine detail—often the best compromise when downstream metrology or boundary localization is critical. The local-contrast maps corroborate these observations by decoupling textural gain from edge sharpness, clarifying the trade-off negotiated by ℓ. Computationally, both selection paths are lightweight: the NPS-based bisection converges in a few iterations, and the (*f*, *g*) scans can be evaluated on a log-spaced grid in minutes on a CPU, with straightforward opportunities for parallel/GPU acceleration.

The proposed methodology is simple to implement and robust to moderate variations in both signal-to-noise ratio and acquisition geometry. It integrates naturally with standard reconstruction pipelines, carrying the projection-level benefits into the volume domain without excessive blurring or artifact amplification. Limitations include the implicit assumption of isotropic, stationary noise and the lack of an explicit MTF model; both can be addressed in future work by incorporating angle-dependent ring partitioning, MTF-aware normalization, or adaptive 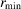 selection. Finally, the accompanying open-source implementation codes (see Appendix *C*[App appc]) provide a transparent path to adoption and benchmarking, supporting routine, objective parameter selection in synchrotron-based tomographic workflows.

## Figures and Tables

**Table 1:** Estimated values of the Paganin-filter parameter ℓ, obtained using the two methods proposed in this work for determining the near-optimal ℓ in equation (1)

Sample (*a*)	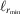 = 381.84 m^2^	ℓ_std_ = 62.65 m^2^	ℓ_corr_ = 62.70 m^2^
Sample (*b*)	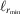 = 2402.81 m^2^	ℓ_std_ = 304.39 m^2^	ℓ_corr_ = 540.35 m^2^
Sample (*c*)	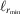 = 3203.74 m^2^	ℓ_std_ = 42.18 m^2^	ℓ_corr_ = 75.72 m^2^

## Appendix A Optimal existence

Let *f*(ℓ) be defined in equation (9) and consider  =  with *P*_ℓ_ = (*I* − ℓΔ)^−1^. By defining *u*_ℓ_ := *P*_ℓ_(*r*) we can compute the derivative of *p*_ℓ_ with respect to ℓ through

For linear steps *h* > 0, we define

so that by Taylor expansion we obtain

It follows therefore that

that is, for fixed and nonzero *h*, the value of *f*_*h*_(0) is of order *h*. In the limit *h* → 0, we have *f*_*h*_ → 0. On the other hand, from equation (13), we know that ∥(*I* − ℓΔ)^−1^Δ∥ ≤ 1/ℓ, so that

implies

for some bounding constant *C*. It then follows that, when ℓ → ∞, we can say

Since *f*_*h*_ is continuous in ℓ ≥ 0 (as it involves a set of continuous operations), and reaches null values at the extremes of the interval [0, ∞), it follows that *f*_*h*_ necessarily attains a local maximum in this interval. Moreover, as *f* ≃ *f*_*h*_ for small *h*, the same behavior of *f*_*h*_ can be transferred to the function *f*, so that it also attains a local maximum in the interval [0, ∞).

## Appendix B Cosine similarity

Let ϕ(ℓ) = *p*_ℓ_/∥*p*_ℓ_∥_2_. To define the approximation in equation (11), it is enough to note that

Thus, by applying the Taylor expansion of ϕ in ℓ, and using the fact that 〈ϕ, ϕ〉 = 1 and 〈ϕ, ϕ′〉 = 0, we obtain

from which the proposed approximation follows. It is worth noting that this approximation is also referred to as ‘cosine similarity’, for obvious reasons.

## Appendix C Python codes

The codes implementing the theoretical framework presented in this manuscript are included here for completeness and to ensure full scientific reproducibility. They are concise and self-contained, being designed so as not to interrupt the flow or clarity of the main text. The Python function <optimal_nps>, shown in Code 1[Chem scheme1a], implements the computation of the near-optimal value —as defined by the criterion in equation (7)—for a given input parameter *r*_min_, together with the normalized image *r* = *I*/*I*_0_, where *I*_0_ denotes the measured flat-field and *I* denotes the corresponding transmission recorded in the experiment. For the datasets analyzed in this work, *r* corresponds to a real-valued image of 2048 × 2048 pixels. On a standard CPU workstation, the calculation of  takes approximately 40 s on average, demonstrating that the procedure is computationally lightweight and practical for routine tomographic data processing.[Chem scheme1a][Chem scheme1b]

The Python function <optimal_std_corr>, presented in Code 2, implements the computation of the near-optimal value of ℓ_std_, ℓ_corr_ according to equations (9) and (11). This implementation follows directly from the theoretical formulation introduced in Section 2, providing a practical tool to determine ℓ through the joint evaluation of the standard-deviation and correlation criteria. The procedure scans the parameter range defined by  and identifies the maximum and minimum of the merit functions *f*(ℓ) and *g*(ℓ), respectively, thereby yielding ℓ_std_ and ℓ_corr_. The average execution time of the presented implementation is approximately 89 s when run solely on a CPU, without any parallelization or hardware acceleration. Despite this modest computational cost, the method remains efficient and reproducible, and can be further accelerated through straightforward parallelization strategies.[Chem scheme2]
